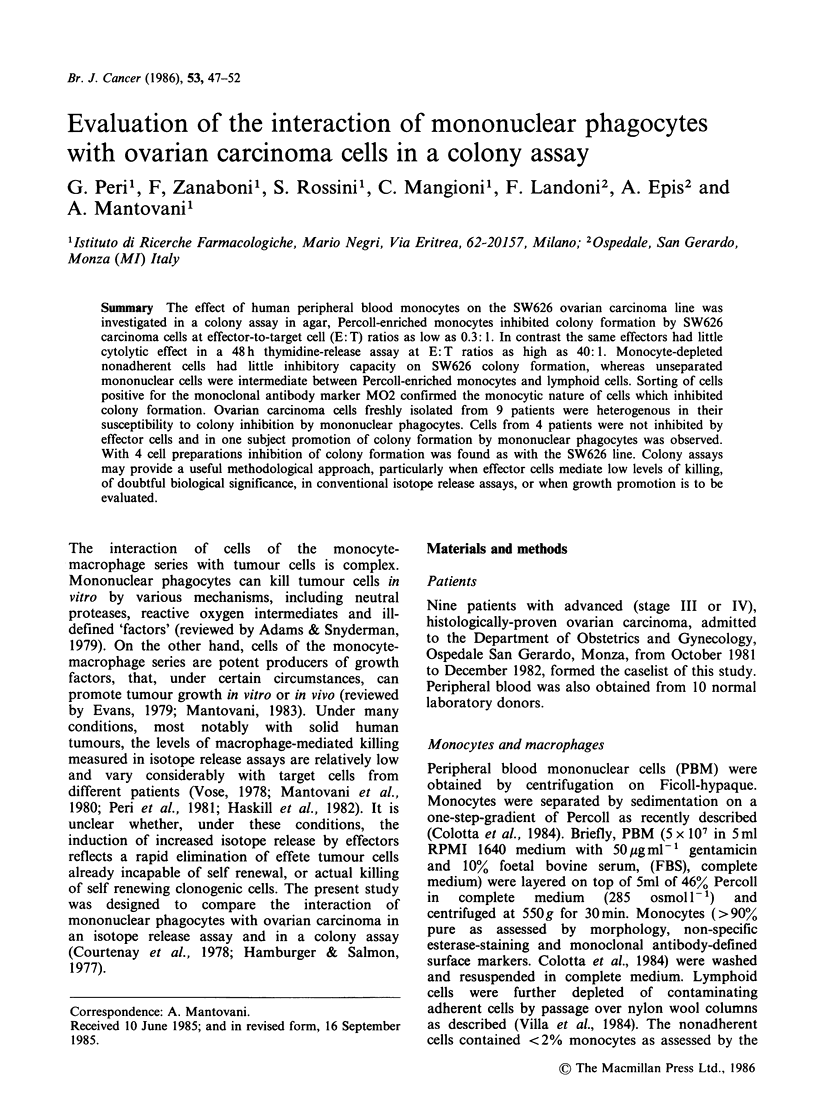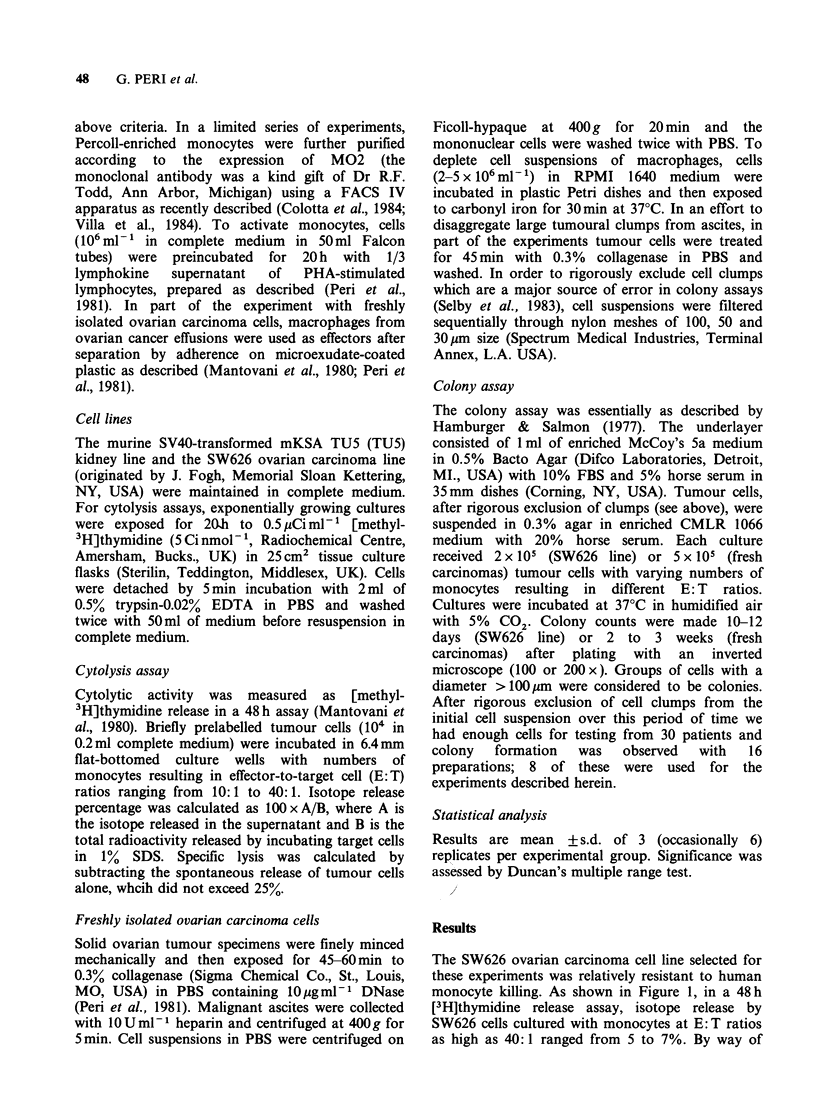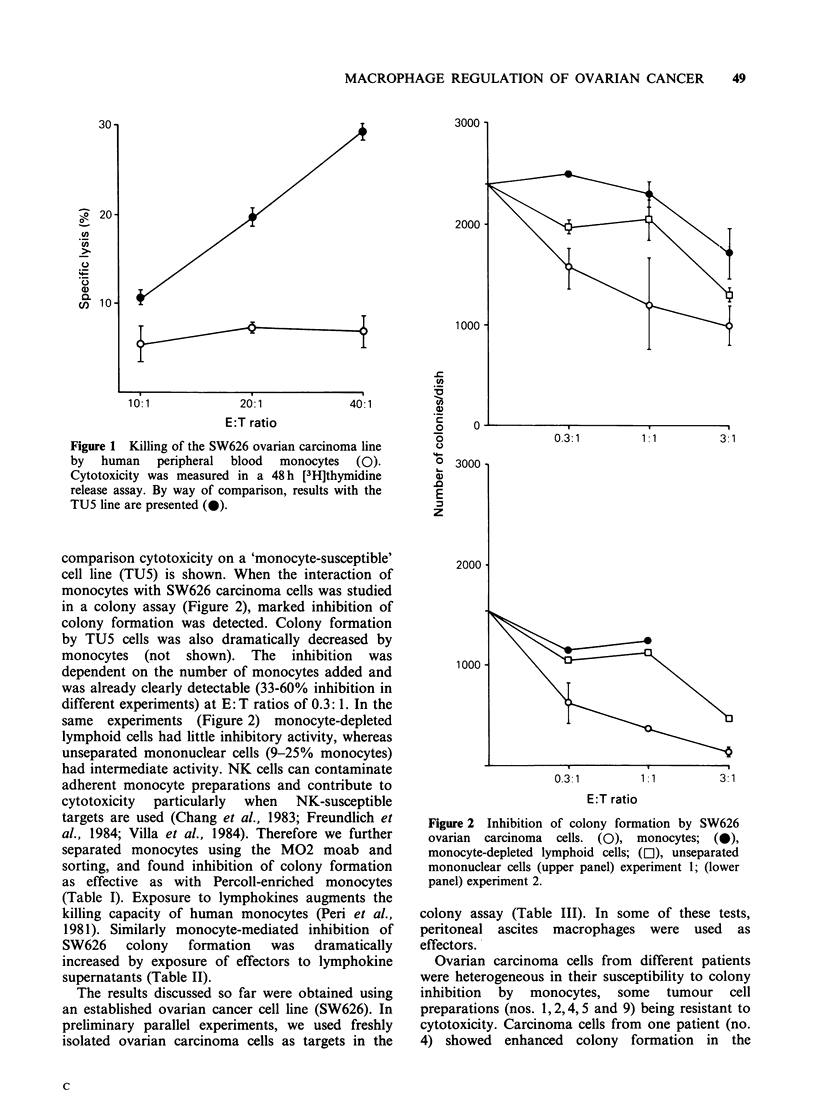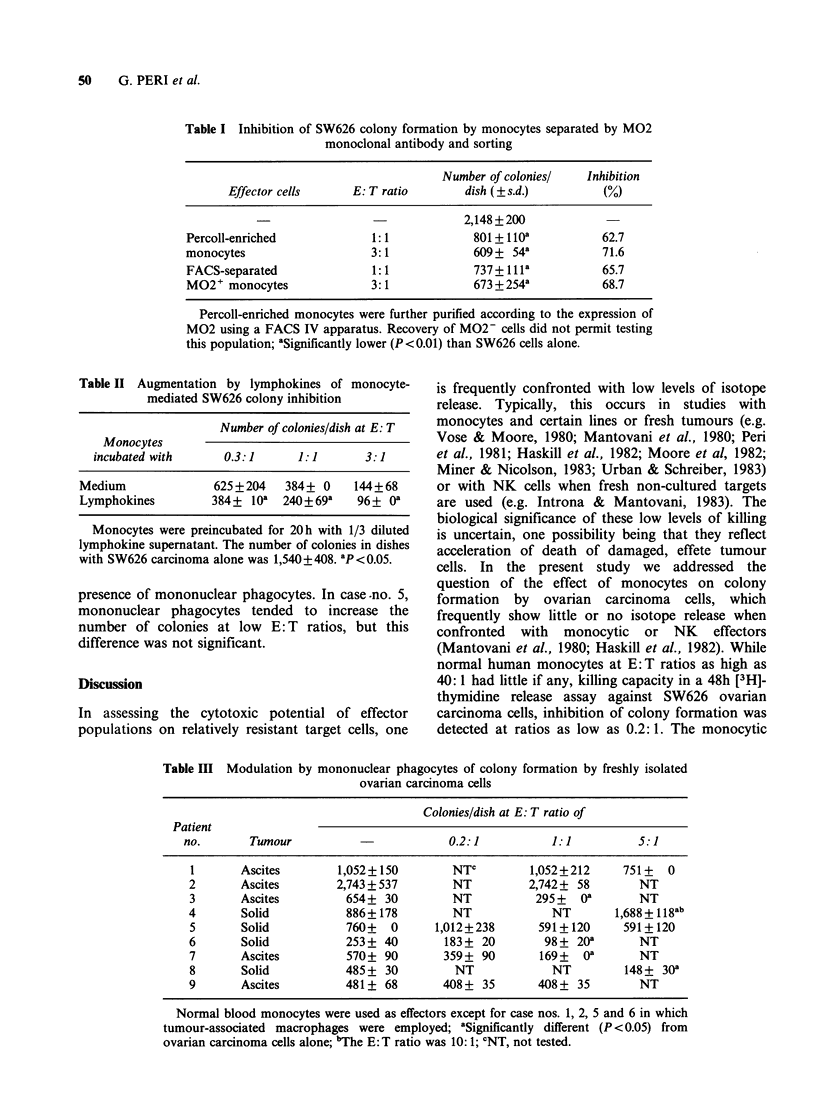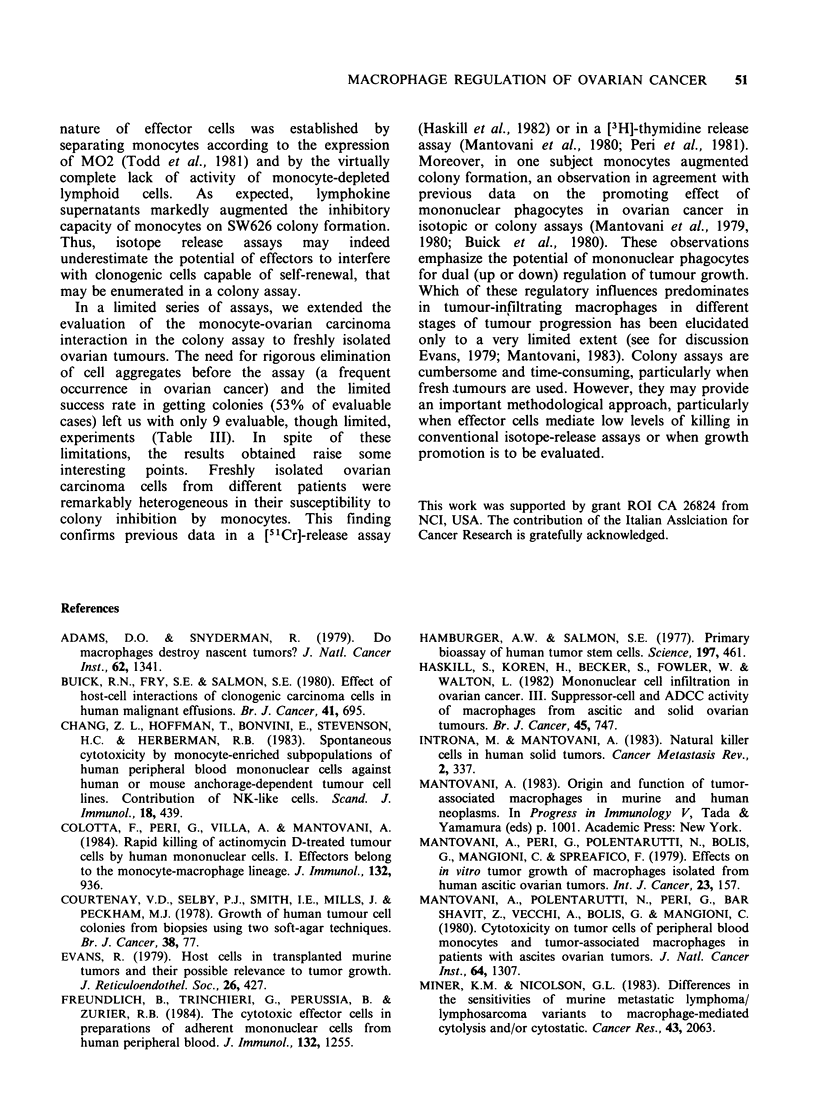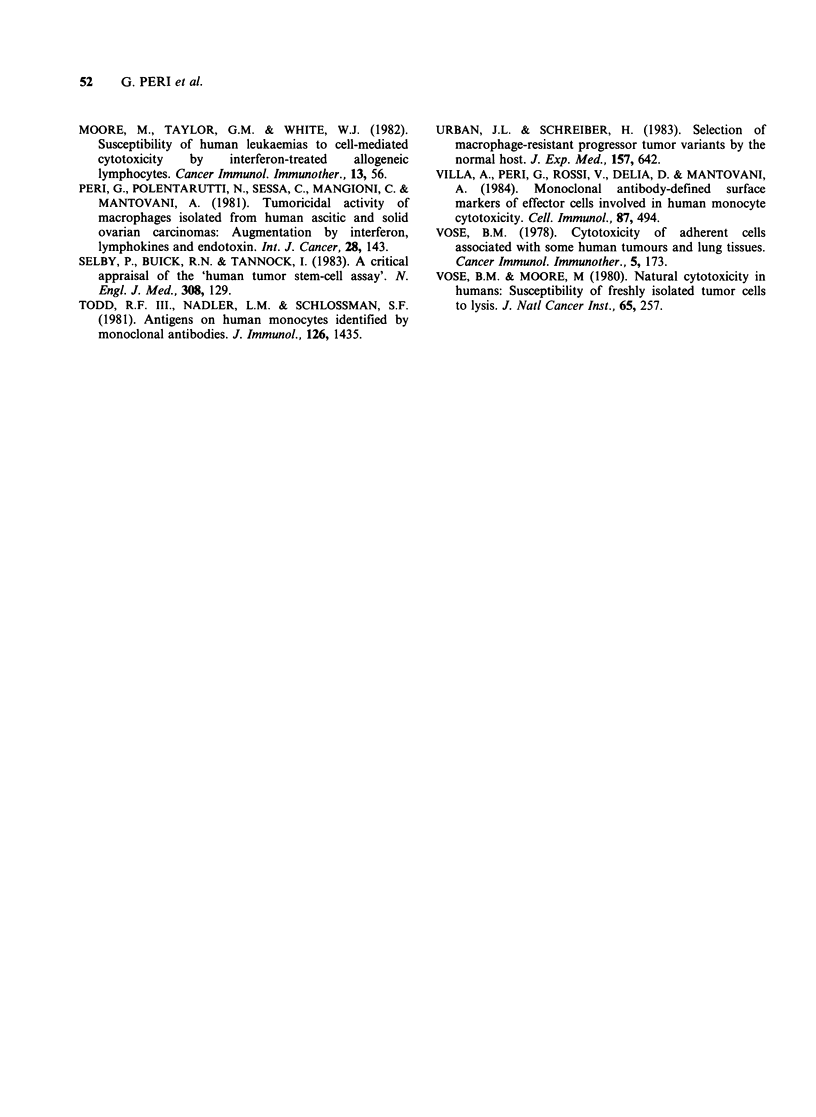# Evaluation of the interaction of mononuclear phagocytes with ovarian carcinoma cells in a colony assay.

**DOI:** 10.1038/bjc.1986.7

**Published:** 1986-01

**Authors:** G. Peri, F. Zanaboni, S. Rossini, C. Mangioni, F. Landoni, A. Epis, A. Mantovani

## Abstract

The effect of human peripheral blood monocytes on the SW626 ovarian carcinoma line was investigated in a colony assay in agar, Percoll-enriched monocytes inhibited colony formation by SW626 carcinoma cells at effector-to-target cell (E:T) ratios as low as 0.3:1. In contrast the same effectors had little cytolytic effect in a 48 h thymidine-release assay at E:T ratios as high as 40:1. Monocyte-depleted nonadherent cells had little inhibitory capacity on SW626 colony formation, whereas unseparated mononuclear cells were intermediate between Percoll-enriched monocytes and lymphoid cells. Sorting of cells positive for the monoclonal antibody marker MO2 confirmed the monocytic nature of cells which inhibited colony formation. Ovarian carcinoma cells freshly isolated from 9 patients were heterogenous in their susceptibility to colony inhibition by mononuclear phagocytes. Cells from 4 patients were not inhibited by effector cells and in one subject promotion of colony formation by mononuclear phagocytes was observed. With 4 cell preparations inhibition of colony formation was found as with the SW626 line. Colony assays may provide a useful methodological approach, particularly when effector cells mediate low levels of killing, of doubtful biological significance, in conventional isotope release assays, or when growth promotion is to be evaluated.


*Br. J. Cancer* (1986), **53**, 47–52

### Evaluation of the interaction of mononuclear phagocytes with ovarian carcinoma cells in a colony assay

G. Peri[1], F, Zanaboni[1], S. Rossini[1], C. Mangioni[1], F. Landoni[2], A. Epis[2] and A. Mantovani[1]

[1]*Istituto di Ricerche Farmacologiche, Mario Negri, Via Eritrea, 62-20157, Milano;* [2]*Ospedale, San Gerardo, Monza (MI) Italy*

**Summary** The effect of human peripheral blood monocytes on the SW626 ovarian carcinoma line was investigated in a colony assay in agar, Percoll-enriched monocytes inhibited colony formation by SW626 carcinoma cells at effector-to-target cell (E:T) ratios as low as 0.3:1. In contrast the same effectors had little cytolytic effect in a 48 h thymidine-release assay at E:T ratios as high as 40:1. Monocyte-depleted nonadherent cells had little inhibitory capacity on SW626 colony formation, whereas unseparated mononuclear cells were intermediate between Percoll-enriched monocytes and lymphoid cells. Sorting of cells positive for the monoclonal antibody marker MO2 confirmed the monocytic nature of cells which inhibited colony formation. Ovarian carcinoma cells freshly isolated from 9 patients were heterogenous in their susceptibility to colony inhibition by mononuclear phagocytes. Cells from 4 patients were not inhibited by effector cells and in one subject promotion of colony formation by mononuclear phagocytes was observed. With 4 cell preparations inhibition of colony formation was found as with the SW626 line. Colony assays may provide a useful methodological approach, particularly when effector cells mediate low levels of killing, of doubtful biological significance, in conventional isotope release assays, or when growth promotion is to be evaluated.

The interaction of cells of the monocyte-macrophage series with tumour cells is complex. Mononuclear phagocytes can kill tumour cells *in vitro* by various mechanisms, including neutral proteases, reactive oxygen intermediates and ill-defined 'factors' (reviewed by Adams & Snyderman, 1979). On the other hand, cells of the monocyte-macrophage series are potent producers of growth factors, that, under certain circumstances, can promote tumour growth *in vitro* or *in vivo* (reviewed by Evans, 1979; Mantovani, 1983). Under many conditions, most notably with solid human tumours, the levels of macrophage-mediated killing measured in isotope release assays are relatively low and vary considerably with target cells from different patients (Vose, 1978; Mantovani *et al.*, 1980; Peri *et al.*, 1981; Haskill *et al.*, 1982). It is unclear whether, under these conditions, the induction of increased isotope release by effectors reflects a rapid elimination of effete tumour cells already incapable of self renewal, or actual killing of self renewing clonogenic cells. The present study was designed to compare the interaction of mononuclear phagocytes with ovarian carcinoma in an isotope release assay and in a colony assay (Courtenay *et al.*, 1978; Hamburger & Salmon, 1977).

Correspondence: A. Mantovani.
Received 10 June 1985; and in revised form, 16 September 1985.

### Materials and methods

### *Patients*

Nine patients with advanced (stage III or IV), histologically-proven ovarian carcinoma, admitted to the Department of Obstetrics and Gynecology, Ospedale San Gerardo, Monza, from October 1981 to December 1982, formed the caselist of this study. Peripheral blood was also obtained from 10 normal laboratory donors.

### *Monocytes and macrophages*

Peripheral blood mononuclear cells (PBM) were obtained by centrifugation on Ficoll-hypaque. Monocytes were separated by sedimentation on a one-step-gradient of Percoll as recently described (Colotta *et al.*, 1984). Briefly, PBM ($5\times10^7$ in 5 ml RPMI 1640 medium with $50\,\mu g\,ml^{-1}$ gentamicin and 10% foetal bovine serum, (FBS), complete medium) were layered on top of 5ml of 46% Percoll in complete medium (285 $osmol\,l^{-1}$) and centrifuged at 550 *g* for 30 min. Monocytes (>90% pure as assessed by morphology, non-specific esterase-staining and monoclonal antibody-defined surface markers. Colotta *et al.*, 1984) were washed and resuspended in complete medium. Lymphoid cells were further depleted of contaminating adherent cells by passage over nylon wool columns as described (Villa *et al.*, 1984). The nonadherent cells contained <2% monocytes as assessed by the

© The Macmillan Press Ltd., 1986

above criteria. In a limited series of experiments, Percoll-enriched monocytes were further purified according to the expression of MO2 (the monoclonal antibody was a kind gift of Dr R.F. Todd, Ann Arbor, Michigan) using a FACS IV apparatus as recently described (Colotta *et al.*, 1984; Villa et al., 1984). To activate monocytes, cells ($10^6 ml^{-1}$ in complete medium in 50 ml Falcon tubes) were preincubated for 20 h with 1/3 lymphokine supernatant of PHA-stimulated lymphocytes, prepared as described (Peri *et al.*, 1981). In part of the experiment with freshly isolated ovarian carcinoma cells, macrophages from ovarian cancer effusions were used as effectors after separation by adherence on microexudate-coated plastic as described (Mantovani *et al.*, 1980; Peri *et al.*, 1981).

*Cell lines*

The murine SV40-transformed mKSA TU5 (TU5) kidney line and the SW626 ovarian carcinoma line (originated by J. Fogh, Memorial Sloan Kettering, NY, USA) were maintained in complete medium. For cytolysis assays, exponentially growing cultures were exposed for 20 h to $0.5\,\mu Ci\,ml^{-1}$ [methyl-$^3$H]thymidine ($5\,Ci\,nmol^{-1}$, Radiochemical Centre, Amersham, Bucks., UK) in $25\,cm^2$ tissue culture flasks (Sterilin, Teddington, Middlesex, UK). Cells were detached by 5 min incubation with 2 ml of 0.5% trypsin-0.02% EDTA in PBS and washed twice with 50 ml of medium before resuspension in complete medium.

*Cytolysis assay*

Cytolytic activity was measured as [methyl-$^3$H]thymidine release in a 48 h assay (Mantovani *et al.*, 1980). Briefly prelabelled tumour cells ($10^4$ in 0.2 ml complete medium) were incubated in 6.4 mm flat-bottomed culture wells with numbers of monocytes resulting in effector-to-target cell (E:T) ratios ranging from 10:1 to 40:1. Isotope release percentage was calculated as $100 \times A/B$, where A is the isotope released in the supernatant and B is the total radioactivity released by incubating target cells in 1% SDS. Specific lysis was calculated by subtracting the spontaneous release of tumour cells alone, whcih did not exceed 25%.

*Freshly isolated ovarian carcinoma cells*

Solid ovarian tumour specimens were finely minced mechanically and then exposed for 45–60 min to 0.3% collagenase (Sigma Chemical Co., St., Louis, MO, USA) in PBS containing $10\,\mu g\,ml^{-1}$ DNase (Peri *et al.*, 1981). Malignant ascites were collected with $10\,U\,ml^{-1}$ heparin and centrifuged at 400 *g* for 5 min. Cell suspensions in PBS were centrifuged on Ficoll-hypaque at 400 *g* for 20 min and the mononuclear cells were washed twice with PBS. To deplete cell suspensions of macrophages, cells ($2–5 \times 10^6 ml^{-1}$) in RPMI 1640 medium were incubated in plastic Petri dishes and then exposed to carbonyl iron for 30 min at 37°C. In an effort to disaggregate large tumoural clumps from ascites, in part of the experiments tumour cells were treated for 45 min with 0.3% collagenase in PBS and washed. In order to rigorously exclude cell clumps which are a major source of error in colony assays (Selby *et al.*, 1983), cell suspensions were filtered sequentially through nylon meshes of 100, 50 and 30 μm size (Spectrum Medical Industries, Terminal Annex, L.A. USA).

*Colony assay*

The colony assay was essentially as described by Hamburger & Salmon (1977). The underlayer consisted of 1 ml of enriched McCoy's 5a medium in 0.5% Bacto Agar (Difco Laboratories, Detroit, MI., USA) with 10% FBS and 5% horse serum in 35 mm dishes (Corning, NY, USA). Tumour cells, after rigorous exclusion of clumps (see above), were suspended in 0.3% agar in enriched CMLR 1066 medium with 20% horse serum. Each culture received $2 \times 10^5$ (SW626 line) or $5 \times 10^5$ (fresh carcinomas) tumour cells with varying numbers of monocytes resulting in different E:T ratios. Cultures were incubated at 37°C in humidified air with 5% $CO_2$. Colony counts were made 10–12 days (SW626 line) or 2 to 3 weeks (fresh carcinomas) after plating with an inverted microscope (100 or 200×). Groups of cells with a diameter >100 μm were considered to be colonies. After rigorous exclusion of cell clumps from the initial cell suspension over this period of time we had enough cells for testing from 30 patients and colony formation was observed with 16 preparations; 8 of these were used for the experiments described herein.

*Statistical analysis*

Results are mean ±s.d. of 3 (occasionally 6) replicates per experimental group. Significance was assessed by Duncan's multiple range test.

### Results

The SW626 ovarian carcinoma cell line selected for these experiments was relatively resistant to human monocyte killing. As shown in Figure 1, in a 48 h [$^3$H]thymidine release assay, isotope release by SW626 cells cultured with monocytes at E:T ratios as high as 40:1 ranged from 5 to 7%. By way of

**Figure 1** Killing of the SW626 ovarian carcinoma line by human peripheral blood monocytes (○). Cytotoxicity was measured in a 48 h [$^3$H]thymidine release assay. By way of comparison, results with the TU5 line are presented (●).

comparison cytotoxicity on a 'monocyte-susceptible' cell line (TU5) is shown. When the interaction of monocytes with SW626 carcinoma cells was studied in a colony assay (Figure 2), marked inhibition of colony formation was detected. Colony formation by TU5 cells was also dramatically decreased by monocytes (not shown). The inhibition was dependent on the number of monocytes added and was already clearly detectable (33-60% inhibition in different experiments) at E:T ratios of 0.3:1. In the same experiments (Figure 2) monocyte-depleted lymphoid cells had little inhibitory activity, whereas unseparated mononuclear cells (9–25% monocytes) had intermediate activity. NK cells can contaminate adherent monocyte preparations and contribute to cytotoxicity particularly when NK-susceptible targets are used (Chang *et al.*, 1983; Freundlich *et al.*, 1984; Villa *et al.*, 1984). Therefore we further separated monocytes using the MO2 moab and sorting, and found inhibition of colony formation as effective as with Percoll-enriched monocytes (Table I). Exposure to lymphokines augments the killing capacity of human monocytes (Peri *et al.*, 1981). Similarly monocyte-mediated inhibition of SW626 colony formation was dramatically increased by exposure of effectors to lymphokine supernatants (Table II).

The results discussed so far were obtained using an established ovarian cancer cell line (SW626). In preliminary parallel experiments, we used freshly isolated ovarian carcinoma cells as targets in the

**Figure 2** Inhibition of colony formation by SW626 ovarian carcinoma cells. (○), monocytes; (●), monocyte-depleted lymphoid cells; (□), unseparated mononuclear cells (upper panel) experiment 1; (lower panel) experiment 2.

colony assay (Table III). In some of these tests, peritoneal ascites macrophages were used as effectors.

Ovarian carcinoma cells from different patients were heterogeneous in their susceptibility to colony inhibition by monocytes, some tumour cell preparations (nos. 1, 2, 4, 5 and 9) being resistant to cytotoxicity. Carcinoma cells from one patient (no. 4) showed enhanced colony formation in the

**Table I** Inhibition of SW626 colony formation by monocytes separated by MO2 monoclonal antibody and sorting

| *Effector cells* | *E: T ratio* | *Number of colonies/ dish (±s.d.)* | *Inhibition (%)* |
|---|---|---|---|
| — | — | 2,148±200 | — |
| Percoll-enriched | 1:1 | 801±110[a] | 62.7 |
| monocytes | 3:1 | 609± 54[a] | 71.6 |
| FACS-separated | 1:1 | 737±111[a] | 65.7 |
| MO2$^+$ monocytes | 3:1 | 673±254[a] | 68.7 |

Percoll-enriched monocytes were further purified according to the expression of MO2 using a FACS IV apparatus. Recovery of MO2$^-$ cells did not permit testing this population; [a]Significantly lower ($P<0.01$) than SW626 cells alone.

**Table II** Augmentation by lymphokines of monocyte-mediated SW626 colony inhibition

| *Monocytes incubated with* | *Number of colonies/dish at E: T* | | |
|---|---|---|---|
| | *0.3:1* | *1:1* | *3:1* |
| Medium | 625±204 | 384± 0 | 144±68 |
| Lymphokines | 384± 10[a] | 240±69[a] | 96± 0[a] |

Monocytes were preincubated for 20 h with 1/3 diluted lymphokine supernatant. The number of colonies in dishes with SW626 carcinoma alone was 1,540±408. [a]$P<0.05$.

presence of mononuclear phagocytes. In case no. 5, mononuclear phagocytes tended to increase the number of colonies at low E:T ratios, but this difference was not significant.

### Discussion

In assessing the cytotoxic potential of effector populations on relatively resistant target cells, one is frequently confronted with low levels of isotope release. Typically, this occurs in studies with monocytes and certain lines or fresh tumours (e.g. Vose & Moore, 1980; Mantovani *et al.*, 1980; Peri *et al.*, 1981; Haskill *et al.*, 1982; Moore *et al*, 1982; Miner & Nicolson, 1983; Urban & Schreiber, 1983) or with NK cells when fresh non-cultured targets are used (e.g. Introna & Mantovani, 1983). The biological significance of these low levels of killing is uncertain, one possibility being that they reflect acceleration of death of damaged, effete tumour cells. In the present study we addressed the question of the effect of monocytes on colony formation by ovarian carcinoma cells, which frequently show little or no isotope release when confronted with monocytic or NK effectors (Mantovani *et al.*, 1980; Haskill *et al.*, 1982). While normal human monocytes at E:T ratios as high as 40:1 had little if any, killing capacity in a 48h [$^3$H]-thymidine release assay against SW626 ovarian carcinoma cells, inhibition of colony formation was detected at ratios as low as 0.2:1. The monocytic

**Table III** Modulation by mononuclear phagocytes of colony formation by freshly isolated ovarian carcinoma cells

| *Patient no.* | *Tumour* | *Colonies/dish at E: T ratio of* | | | |
|---|---|---|---|---|---|
| | | — | *0.2:1* | *1:1* | *5:1* |
| 1 | Ascites | 1,052±150 | NT[c] | 1,052±212 | 751± 0 |
| 2 | Ascites | 2,743±537 | NT | 2,742± 58 | NT |
| 3 | Ascites | 654± 30 | NT | 295± 0[a] | NT |
| 4 | Solid | 886±178 | NT | NT | 1,688±118[ab] |
| 5 | Solid | 760± 0 | 1,012±238 | 591±120 | 591±120 |
| 6 | Solid | 253± 40 | 183± 20 | 98± 20[a] | NT |
| 7 | Ascites | 570± 90 | 359± 90 | 169± 0[a] | NT |
| 8 | Solid | 485± 30 | NT | NT | 148± 30[a] |
| 9 | Ascites | 481± 68 | 408± 35 | 408± 35 | NT |

Normal blood monocytes were used as effectors except for case nos. 1, 2, 5 and 6 in which tumour-associated macrophages were employed; [a]Significantly different ($P<0.05$) from ovarian carcinoma cells alone; [b]The E:T ratio was 10:1; [c]NT, not tested.

nature of effector cells was established by separating monocytes according to the expression of MO2 (Todd *et al.*, 1981) and by the virtually complete lack of activity of monocyte-depleted lymphoid cells. As expected, lymphokine supernatants markedly augmented the inhibitory capacity of monocytes on SW626 colony formation. Thus, isotope release assays may indeed underestimate the potential of effectors to interfere with clonogenic cells capable of self-renewal, that may be enumerated in a colony assay.

In a limited series of assays, we extended the evaluation of the monocyte-ovarian carcinoma interaction in the colony assay to freshly isolated ovarian tumours. The need for rigorous elimination of cell aggregates before the assay (a frequent occurrence in ovarian cancer) and the limited success rate in getting colonies (53% of evaluable cases) left us with only 9 evaluable, though limited, experiments (Table III). In spite of these limitations, the results obtained raise some interesting points. Freshly isolated ovarian carcinoma cells from different patients were remarkably heterogeneous in their susceptibility to colony inhibition by monocytes. This finding confirms previous data in a [$^{51}$Cr]-release assay (Haskill *et al.*, 1982) or in a [$^{3}$H]-thymidine release assay (Mantovani *et al.*, 1980; Peri *et al.*, 1981). Moreover, in one subject monocytes augmented colony formation, an observation in agreement with previous data on the promoting effect of mononuclear phagocytes in ovarian cancer in isotopic or colony assays (Mantovani *et al.*, 1979, 1980; Buick *et al.*, 1980). These observations emphasize the potential of mononuclear phagocytes for dual (up or down) regulation of tumour growth. Which of these regulatory influences predominates in tumour-infiltrating macrophages in different stages of tumour progression has been elucidated only to a very limited extent (see for discussion Evans, 1979; Mantovani, 1983). Colony assays are cumbersome and time-consuming, particularly when fresh tumours are used. However, they may provide an important methodological approach, particularly when effector cells mediate low levels of killing in conventional isotope-release assays or when growth promotion is to be evaluated.

This work was supported by grant ROI CA 26824 from NCI, USA. The contribution of the Italian Asslciation for Cancer Research is gratefully acknowledged.

### References

ADAMS, D.O. & SNYDERMAN, R. (1979). Do macrophages destroy nascent tumors? *J. Natl. Cancer Inst.*, **62**, 1341.

BUICK, R.N., FRY, S.E. & SALMON, S.E. (1980). Effect of host-cell interactions of clonogenic carcinoma cells in human malignant effusions. *Br. J. Cancer*, **41**, 695.

CHANG, Z. L., HOFFMAN, T., BONVINI, E., STEVENSON, H.C. & HERBERMAN, R.B. (1983). Spontaneous cytotoxicity by monocyte-enriched subpopulations of human peripheral blood mononuclear cells against human or mouse anchorage-dependent tumour cell lines. Contribution of NK-like cells. *Scand. J. Immunol.*, **18**, 439.

COLOTTA, F., PERI, G., VILLA, A. & MANTOVANI, A. (1984). Rapid killing of actinomycin D-treated tumour cells by human mononuclear cells. I. Effectors belong to the monocyte-macrophage lineage. *J. Immunol.*, **132**, 936.

COURTENAY, V.D., SELBY, P.J., SMITH, I.E., MILLS, J. & PECKHAM, M.J. (1978). Growth of human tumour cell colonies from biopsies using two soft-agar techniques. *Br. J. Cancer*, **38**, 77.

EVANS, R. (1979). Host cells in transplanted murine tumors and their possible relevance to tumor growth. *J. Reticuloendothel. Soc.*, **26**, 427.

FREUNDLICH, B., TRINCHIERI, G., PERUSSIA, B. & ZURIER, R.B. (1984). The cytotoxic effector cells in preparations of adherent mononuclear cells from human peripheral blood. *J. Immunol.*, **132**, 1255.

HAMBURGER, A.W. & SALMON, S.E. (1977). Primary bioassay of human tumor stem cells. *Science*, **197**, 461.

HASKILL, S., KOREN, H., BECKER, S., FOWLER, W. & WALTON, L. (1982) Mononuclear cell infiltration in ovarian cancer. III. Suppressor-cell and ADCC activity of macrophages from ascitic and solid ovarian tumours. *Br. J. Cancer*, **45**, 747.

INTRONA, M. & MANTOVANI, A. (1983). Natural killer cells in human solid tumors. *Cancer Metastasis Rev.*, **2**, 337.

MANTOVANI, A. (1983). Origin and function of tumor-associated macrophages in murine and human neoplasms. In *Progress in Immunology V*, Tada & Yamamura (eds) p. 1001. Academic Press: New York.

MANTOVANI, A., PERI, G., POLENTARUTTI, N., BOLIS, G., MANGIONI, C. & SPREAFICO, F. (1979). Effects on *in vitro* tumor growth of macrophages isolated from human ascitic ovarian tumors. *Int. J. Cancer*, **23**, 157.

MANTOVANI, A., POLENTARUTTI, N., PERI, G., BAR SHAVIT, Z., VECCHI, A., BOLIS, G. & MANGIONI, C. (1980). Cytotoxicity on tumor cells of peripheral blood monocytes and tumor-associated macrophages in patients with ascites ovarian tumors. *J. Natl. Cancer Inst.*, **64**, 1307.

MINER, K.M. & NICOLSON, G.L. (1983). Differences in the sensitivities of murine metastatic lymphoma/lymphosarcoma variants to macrophage-mediated cytolysis and/or cytostatic. *Cancer Res.*, **43**, 2063.

MOORE, M., TAYLOR, G.M. & WHITE, W.J. (1982). Susceptibility of human leukaemias to cell-mediated cytotoxicity by interferon-treated allogeneic lymphocytes. *Cancer Immunol. Immunother.*, **13**, 56.

PERI, G., POLENTARUTTI, N., SESSA, C., MANGIONI, C. & MANTOVANI, A. (1981). Tumoricidal activity of macrophages isolated from human ascitic and solid ovarian carcinomas: Augmentation by interferon, lymphokines and endotoxin. *Int. J. Cancer*, **28**, 143.

SELBY, P., BUICK, R.N. & TANNOCK, I. (1983). A critical appraisal of the 'human tumor stem-cell assay'. *N. Engl. J. Med.*, **308**, 129.

TODD, R.F. III., NADLER, L.M. & SCHLOSSMAN, S.F. (1981). Antigens on human monocytes identified by monoclonal antibodies. *J. Immunol.*, **126**, 1435.

URBAN, J.L. & SCHREIBER, H. (1983). Selection of macrophage-resistant progressor tumor variants by the normal host. *J. Exp. Med.*, **157**, 642.

VILLA, A., PERI, G., ROSSI, V., DELIA, D. & MANTOVANI, A. (1984). Monoclonal antibody-defined surface markers of effector cells involved in human monocyte cytotoxicity. *Cell. Immunol.*, **87**, 494.

VOSE, B.M. (1978). Cytotoxicity of adherent cells associated with some human tumours and lung tissues. *Cancer Immunol. Immunother.*, **5**, 173.

VOSE, B.M. & MOORE, M (1980). Natural cytotoxicity in humans: Susceptibility of freshly isolated tumor cells to lysis. *J. Natl Cancer Inst.*, **65**, 257.